# Dataset of energetically accessible structures of MgCl_2_/TiCl_4_ clusters for Ziegler-Natta catalysts

**DOI:** 10.1016/j.dib.2020.106654

**Published:** 2020-12-15

**Authors:** Gentoku Takasao, Toru Wada, Ashutosh Thakur, Patchanee Chammingkwan, Minoru Terano, Toshiaki Taniike

**Affiliations:** a Graduate School of Advanced Science and Technology, Japan Advanced Institute of Science and Technology, 1-1 Asahidai, Nomi, Ishikawa 923-1292, Japan; b DPI, P.O. Box 902, 5600 AX, Eindhoven, the Netherlands

**Keywords:** Ziegler-Natta catalyst, Genetic algorithm, Structure determination, Density functional theory, MgCl_2_, TiCl_4_

## Abstract

This data article provides a dataset of the energetically accessible structures including the most stable structures of *x*MgCl_2_/*y*TiCl_4_ nanoplates (*x* = 6–19, *y* = 0–4). TiCl_4_-capped MgCl_2_ nanoplates are regarded as the building block of the Ziegler–Natta catalyst. The most stable structures were determined for MgCl_2_/TiCl_4_ nanoplates of different sizes and chemical compositions using a combination of the genetic algorithm and the DFT geometry optimization. The evolution in the genetic algorithm produced a number of meta-stable structures. A set of isomeric structures having similar energy to the most stable structure (termed energetically accessible structures) are provided as realistic models of MgCl_2_/TiCl_4_ nanoplates. These structures are useful for further investigation on the structural distribution of Ti species on MgCl_2_ regarding the Ziegler-Natta catalyst.

## Specifications Table

**Table:** 

Subject	Catalysis
Specific subject area	Heterogeneous Ziegler-Natta catalyst
Type of data	A collection of geometry-optimized atom coordinates in the cartesian coordinate file (.car) format in angstroms
How data were acquired	Materials Studio DMol^3^ version 2017
Data format	Raw and Filtered
Parameters for data collection	Perdew–Burke–Ernzerhof (PBE) GGA exchange-correlation functional Double-numerical basis set with polarization functions (DNP) with effective core potential
Description of data collection	722 structure models of *x*MgCl_2_/*y*TiCl_4_ nanoplates (*x* = 6–19, *y* = 0–4) obtained through non-empirical structure determination based on a combination of the genetic algorithm and the DFT geometry optimization.
Data source location	Graduate School of Advanced Science and Technology, Japan Advanced Institute of Science and Technology, Nomi, Ishikawa, Japan
Data accessibility	Repository name: Mendeley Data Data identification number: DOI: 10.17632/c2cv8pg5d8.2 Direct URL to data: https://data.mendeley.com/datasets/c2cv8pg5d8/2
Related research article	Gentoku Takasao, Toru Wada, Ashutosh Thakur, Patchanee Chammingkwan, Minoru Terano and Toshiaki Taniike, Insight into Structural Distribution of Heterogeneous Ziegler–Natta Catalyst from Non-empirical Structure Determination, J. Catal. In Press. https://doi.org/10.1016/j.jcat.2020.11.005

## Value of the Data

Computational chemistry in the field of Ziegler-Natta catalyst conventionally assumed ideal surfaces of MgCl_2_. However, the building block of the actual catalyst is nano-sized and the resultant non-ideality of surfaces would lead to a structural distribution. Here provided energetically accessible structures for *x*MgCl_2_/*y*TiCl_4_ nanoplates are useful to study such a distribution.

The dataset is useful for researchers in the field of heterogeneous olefin polymerization catalysts.

The dataset provides energetically accessible structures as realistic models of MgCl_2_/TiCl_4_. They could be used to investigate the nature and the distribution of TiCl_4_ (or its activated form) in relation to Ziegler-Natta catalysis. They also withstand models for interpreting experimental spectroscopic observations.

## 1 Data Description

The dataset presented in this article provides energetically accessible structures including the most stable structures for *x*MgCl_2_/*y*TiCl_4_ clusters (*x* = 6–19, *y* = 0–4; [Ti] < 10 wt%). These structures were obtained in the course of the structure determination using a combination of the genetic algorithm and the DFT geometry optimization. Here, energetically accessible structures are termed as the isomeric structures whose energies are not greater than 6 kcal/mol with respect to the most stable structure.

The zip file contains 722 structure files, which are organized into folders according to size and chemical composition. All the structure files (in Angstrom) are provided as cartesian coordinate files (.car) and named as follows: “*x*MgCl2_*y*TiCl4_*z*.car”, where *z* corresponds to the energetic order within the same size and composition.

Absolute energies in Hartree that were computed are summarized in “*x*MgCl2_*y*TiCl4.csv”.

## 2 Experimental Design, Materials and Methods

The dataset in this paper was obtained by non-empirical structure determination for *x*MgCl_2_/*y*TiCl_4_ (*x* = 6–19, *y* = 0–4; [Ti] < 10 wt%) [1]. The program for the non-empirical structure determination was developed in our previous paper [2]. The program particularly targets TiCl_4_-capped MgCl_2_ nanoplates whose lateral surfaces are capped with TiCl_4_ molecules. The energies of structures are determined by DFT geometry optimization, and they are used to updates the structures to lower the energy. The program collects newly generated structures to a database in the process of evolution without allowing registration of redundant structures. Further details of the program are given in the literature [2].

The structure determination was performed until the solution satisfied the requirement that multiple runs independently converged to the same physicochemically reasonable structure after a sufficient number of generations. The solution thus obtained by the genetic algorithm was regarded as the optimal one, i.e. the most stable structure.

The size of 6–19MgCl_2_ was chosen with a consideration of a balance between computational feasibility and actuality. 19MgCl_2_, which has a size of about 1.5 nm, was not far from the experimentally determined size of primary particles (ca. 2.4–4.0 nm) [3], [4], [5], [6]. The number of TiCl_4_ molecules ([Ti] < 10 wt%) referred to a typical Ti content of commercial Ziegler–Natta catalysts, which is 1–3 wt% for propylene polymerization and up to 10 wt% for ethylene polymerization [7].

The DFT geometry optimization was performed by DMol^3^ of Materials Studio with the following conditions: The GGA PBE for the exchange-correlation functional [8], and the DNP basis set [9] with effective core potentials [10,11]. The convergence criteria for geometry optimization were set to 0.01255 kcal/mol in energy, 2.510 kcal/(mol Å) in force, and 0.005 Å in displacement. Thermal smearing was used to improve the self-consistent field (SCF) convergence with a value of 0.005 Hartree. The orbital cutoff radius was set to 4.300 Å.

From the structures obtained as described above, the energetically accessible structures were extracted. Here, the word “energetically accessible structures” indicates the isomeric structures (i.e. metastable structures) whose energies are not greater than 6 kcal/mol with respect to the most stable structure.

## Ethics Statement

This work does not involve the use of human subjects and animal experiments.

## CRediT Author Statement

**Gentoku Takasao:** Methodology, Software, Formal Analysis, Data Curation. Writing- Original draft preparation.

**Toru Wada:** Validation, Writing - Review & Editing. **Ashutosh Thakur:** Validation.

**Patchanee Chammingkwan:** Validation. **Minoru Terano:** Validation. **Toshiaki Taniike:** Conceptualization, Supervision.

## Declaration of Competing Interest

The authors declare that they have no known competing financial interests or personal relationships which have, or could be perceived to have, influenced the work reported in this article.
